# A Novel Application of RNA In Situ Hybridization in the Analysis of Vitamin D Receptor Expression in Psoriatic Skin Tissue Following Etanercept Treatment

**DOI:** 10.3390/cimb47050311

**Published:** 2025-04-28

**Authors:** Max Lundgren, Alexandra Kuliszkiewicz, Martin Gillstedt, Azin Jasmin Zanghaneh, Amra Osmancevic

**Affiliations:** 1 Department of Dermatology, Institute of Clinical Sciences, Sahlgrenska Academy, University of Gothenburg, 413 45 Gothenburg, Sweden; max.lundgren@vgregion.se (M.L.); martin.gillstedt@vgregion.se (M.G.); 2 Department of Dermatology and Venereology, Sahlgrenska University Hospital, Region Västra Götaland, 413 45 Gothenburg, Sweden; 3 Department of Physiology and Pharmacology, Karolinska University Hospital, Biomedicum B5, 171 45 Stockholm, Sweden; alexandra.kuliszkiewicz@ki.se; 4 Department of Dermatology and Venereology, Västmanlands Sjukhus Västerås, 721 89 Västerås, Sweden; azin.jasmin.zanghaneh@regionvastmanland.se

**Keywords:** vitamin D, vitamin D receptor, RNAscope, psoriasis, etanercept

## Abstract

Psoriasis is a chronic inflammatory skin disease marked by abnormal keratinocyte proliferation and immune dysregulation. The vitamin D receptor (VDR) plays a crucial role in regulating skin cell growth and immune responses, but its expression in psoriatic skin and modulation by treatment remain unclear. This study aimed to analyze VDR mRNA expression in psoriatic skin tissue before and after etanercept therapy using RNAscope, an RNA in situ hybridization technique that, to the best of our knowledge, has not previously been applied in psoriasis research. Two bio-naïve adult patients with moderate to severe plaque psoriasis received etanercept (50 mg weekly) for 12 weeks. Skin biopsies from lesional and perilesional areas were collected at baseline and post-treatment. VDR expression was assessed in different epidermal layers and the dermis using a semi-quantitative scoring system. In one patient, a statistically significant decrease in VDR expression was observed in the perilesional dermis after treatment (*p* < 0.001), though this preliminary finding warrants careful interpretation given the very limited cohort size. Both patients exhibited a non-significant trend toward increased VDR expression in the lesional epidermis post-treatment. These preliminary findings suggest that etanercept may modulate VDR expression in psoriatic skin, but individual variability and the small sample size preclude definitive conclusions. The study primarily demonstrates the feasibility of using RNAscope for VDR analysis in patients with psoriasis, an approach that may be novel in this context, and underscores the need for larger investigations to confirm these preliminary findings and further clarify the role of VDR in disease pathogenesis and treatment response.

## 1. Introduction

Psoriasis is a chronic inflammatory skin condition that affects approximately 2–3% of the global population, with varying prevalence based on geographic and demographic factors [1]. The most common form of psoriasis, plaque psoriasis, manifests clinically as red, scaly skin patches resulting from the hyperproliferation and abnormal differentiation of keratinocytes, accompanied by a dysregulated immune response. The visible nature of the lesions can lead to significant psychological distress, including anxiety, depression, and social stigmatization [2,3].

Despite extensive research, the molecular pathogenesis of psoriasis is yet to be fully understood [4]. The disease is believed to result from a complex interaction between genetic susceptibility and environmental triggers [5,6]. Genetic predispositions, particularly specific alleles associated with the immune system, contribute to an individual’s susceptibility to the disease [7]. Environmental factors such as skin injuries, infections, stress, and certain medications can initiate or exacerbate the inflammatory response characteristic of psoriasis [5].

Given the central role of immune dysregulation in psoriasis, targeting key inflammatory mediators has become a core treatment strategy. Etanercept, a biologic agent that functions as a soluble tumor necrosis factor-alpha (TNF-α) receptor, is commonly used in the treatment of moderate to severe psoriasis [8]. By acting as a decoy receptor, etanercept binds to circulating TNF-α molecules, preventing their interaction with cell surface TNF receptors and disrupting downstream signaling pathways that contribute to inflammation and immune activation [9,10].

Emerging evidence suggests that vitamin D plays a more significant role in the pathophysiology of psoriasis than previously understood, particularly regarding immune regulation and inflammation [6,11,12]. Through its active form, 1,25-dihydroxyvitamin D (1,25(OH)_2_D), vitamin D modulates excessive immune reactions by downregulating pro-inflammatory cytokines and promoting regulatory T cells, which helps reduce inflammation in psoriasis [13].

Vitamin D also influences various cellular processes via the VDR, including keratinocyte proliferation, differentiation, and apoptosis [14,15]. The biological effects of 1,25(OH)_2_D are mediated through the nuclear VDR, which is heavily expressed in keratinocytes and other skin cells [16]. As such, the skin is not only a site for vitamin D synthesis but also a target organ where vitamin D regulates critical functions affecting skin homeostasis.

The synthesis of vitamin D begins in the skin upon exposure to ultraviolet B (UVB) light, specifically within the 290–315 nm range, converting 7-dehydrocholesterol (7-DHC) in the skin to pre-vitamin D_3_, and then to vitamin D_3_ (cholecalciferol) [13]. Although vitamin D can also be obtained from dietary sources, the skin-produced vitamin D remains the primary source for the body [17].

After synthesis, vitamin D undergoes two hydroxylation steps: first in the liver, where it is converted to 25-hydroxyvitamin D (25(OH)D), and then in the kidneys to its most active form, 1,25(OH)_2_D [13]. This activation process was initially thought to only occur in the kidneys, but it has since been found that extra-renal cells, including keratinocytes and immune cells, also possess the enzyme 1-alpha-hydroxylase necessary for this conversion [18,19].

As such, vitamin D and TNF-α both play important roles in immune modulation. Vitamin D has been shown to downregulate TNF-α expression and activity, exerting anti-inflammatory effects [20]. A previous in vitro study on patients with rheumatoid arthritis demonstrated that while TNF-α blockade does not suppress the production of IL-17A and IL-22, the combination with 1,25(OH)2D could control human Th17 activity and synergistically inhibit synovial inflammation [21]. Similarly, in patients with inflammatory bowel disease, a retrospective cohort study found that those with normal vitamin D levels at the initiation of anti-TNF-α therapy had a 2.64 times increased odds of achieving remission after three months compared to patients with low vitamin D levels, suggesting that adequate vitamin D may enhance the effectiveness of anti-TNF-α treatments [20]. Furthermore, a study by Vandikas et al. on patients with psoriasis treated with etanercept found that those with sufficient levels of serum 25(OH)D at the start of TNF-α inhibitor therapy improved more rapidly on the Visual Analogue Scale (VAS) than those with insufficient levels [22]. These findings suggest that vitamin D status could influence the efficacy of TNF-α inhibitors, highlighting the potential benefits of combining vitamin D optimization with TNF-α inhibition in therapeutic strategies for autoimmune diseases, including psoriasis [20,21,22].

Several studies using immunohistochemistry have reported altered VDR expression in psoriatic lesions. Milde et al. found an increase in VDR expression within psoriatic lesions compared to normal and perilesional skin [23], whereas Kim et al. reported a significant decrease, noting a five-fold reduction [24]. Visconti et al. also observed a reduction in VDR expression, correlating decreased levels with reduced tight junction proteins [25]. Chandra et al. identified a significant negative correlation between VDR expression and both psoriasis severity and disease duration, indicating that VDR levels decrease as the disease progresses [26]. Similarly, Elgarhy et al. demonstrated a significant decrease in VDR expression in psoriatic lesions compared to perilesional skin prior to treatment, which increased after narrow-band UVB therapy [27].

Collectively, these studies suggest that VDR is significantly involved in the pathophysiology of psoriasis. As such, given both the immunomodulatory effects of vitamin D and the therapeutic action of etanercept, exploring the connection between VDR expression and TNF-α inhibition becomes particularly relevant.

The aim of this study was to investigate VDR expression in psoriatic skin tissue before and after etanercept treatment by using RNAscope, a novel RNA in situ hybridization (ISH) technique not previously applied in psoriasis research. This approach enables sensitive, transcript-level analysis in psoriatic lesions, offering new avenues for understanding disease pathogenesis and treatment response.

We hypothesized that etanercept treatment would normalize VDR expression in psoriatic lesions. Assessing these changes could provide insights into how TNF-α inhibition affects vitamin D signaling, thereby deepening our understanding of psoriasis pathophysiology and optimizing future treatment strategies.

## 2. Materials and Methods

### 2.1. Study Design, Setting, and Participants

The study was designed as a prospective intervention study with controls and involved the recruitment of patients from the Department of Dermatology and Venereology at Sahlgrenska University Hospital, Gothenburg, Sweden, during the years 2014–2016. Two adult patients diagnosed with moderate to severe psoriasis and scheduled to receive etanercept treatment were included in the study. Additionally, one healthy control subject was included for comparison (Figure S1).

### 2.2. Inclusion and Exclusion Criteria

Eligible for inclusion were patients over 18 years of age, diagnosed with moderate to severe psoriasis, defined by a Psoriasis Area and Severity Index (PASI) of >10 and/or a Dermatology Life Quality Index (DLQI) of >10, indicating a need for systemic treatment, and who were bio-naïve.

Exclusion criteria included pregnancy, breastfeeding, planning for pregnancy, treatment for severe chronic or systemic illnesses (such as liver or kidney diseases, cancer, infectious diseases), use of oral steroids (local application allowed), other immunosuppressive medications, recent antibiotic treatment, or recent excessive UV light exposure (both artificial and natural), including planned exposure.

Patients with psoriasis treated with etanercept were further excluded if they had active infections (local or systemic), latent tuberculosis, malignancy (except adequately treated non-melanoma skin cancer and malignancies over 10 years ago), demyelinating diseases, heart failure, or a history of more than 200 treatments with psoralen combined with ultraviolet A radiation (PUVA).

### 2.3. Treatment Plan

Patients with psoriasis were treated with etanercept (Enbrel), administered weekly at a dosage of 50 mg subcutaneously for 12 weeks.

Participants attended a total of five visits throughout the study period. Visit 1 involved the collection of baseline skin biopsies from both lesional and perilesional areas prior to treatment, as well as the measurement of PASI and DLQI to establish baseline clinical parameters. Visit 3 (after 10–12 weeks of treatment) included follow-up skin biopsies obtained from the same lesional and perilesional areas to later analyze the treatment’s effect on VDR expression, along with a second measurement of PASI and DLQI to evaluate treatment response. Visits 2, 4, and 5 were reserved for clinical evaluations and monitoring.

### 2.4. Sample Collection

Skin biopsies were collected from each participant at two time points: prior to the initiation of etanercept treatment (Visit 1) and after 10–12 weeks of treatment (Visit 3). At each time point, biopsies were obtained from both lesional and perilesional skin areas. Lesional biopsies were taken from areas of active psoriasis, characterized by erythema, scaling, and plaque formation. Perilesional biopsies were obtained from clinically normal-appearing skin located approximately 2 cm from the lesional site.

To ensure consistency and comparability between pre- and post-treatment samples, biopsies were collected from the same anatomical locations, typically from areas with minimal UV exposure, such as the buttocks.

Each biopsy was taken under local anesthesia using a 4 mm biopsy punch. Following collection, biopsies were immediately placed in cryovials and snap-frozen in liquid nitrogen to preserve tissue integrity and stored at −80 °C until further processing. All biopsy procedures were carried out by the same dermatologist to minimize variability in sample collection.

### 2.5. Preparation of Skin Specimens

Following collection and storage, the biopsies were carefully split in half using a cryostat set to −25 °C. One part of each biopsy was reserved for RNAscope analysis, while the other was allocated for a separate study. The skin biopsies designated for RNAscope were then embedded in optimal cutting temperature (OCT) media within a cryomold. The embedded biopsies were subsequently stored at −80 °C until it was time for the RNAscope staining. When ready for processing, the embedded biopsies relevant to this article were sectioned at a thickness of 10 μm using a cryostat set to −25 °C, resulting in 2 to 6 sections per slide. The number of sections per slide was determined by the size and quality of the biopsy samples, ensuring that sufficient material was available for analysis. This process led to a total of 28 sections across all biopsies, which were subsequently used for RNAscope analysis.

### 2.6. RNAscope Staining Protocol

The RNAscope^®^ 2.5 HD Duplex Chromogenic Assay (Advanced Cell Diagnostics, (ACD), Newark, CA, USA) was utilized to visualize VDR mRNA expression in the skin biopsy sections and performed as instructed by ACD [28].

Skin sections were removed from −80 °C and immediately immersed in pre-chilled 4% paraformaldehyde (PFA) for 15 min at 4 °C. Slides were then removed from PFA and transferred to 50% EtOH and incubated for 5 min at room temperature (RT). This was then repeated using 70% and 100% EtOH (2×). Following air drying at RT for 5 min, the hydrophobic barrier was drawn around the tissue using the ImmEdge™ (Vector Laboratories, Inc., Newark, NJ, USA). Tissue was then incubated with RNAscope Hydrogen Peroxide for 10 min at RT and washed with 1x Phosphate-Buffered Saline (PBS, pH 7.4). Next, sections were incubated with Protease IV (included in the kit) for 30 min at RT, followed by a wash with PBS. RNAscope^®^ 2.5 HD Duplex Chromogenic Assay was performed with pre-warmed probes against human vitamin D receptor (Hh-VDR-C1, Cat. Nr. 530961). After probe hybridization, signal amplification and detection reagents were applied sequentially and incubated in AMP 1, AMP 2, AMP 3, AMP 4, AMP 5, AMP 6 reagents for 30, 15, 30, 15, 30, 15 min, respectively. Before each AMP incubation, samples were washed twice with Wash Buffer (Cat. Nr. 310091, ACD). After detection of the duplex signal, green and red for channels 1 (VDR) and 2, respectively, the slides were counterstained with 50% hematoxylin solution in distilled water and mounted using VectaMount mounting medium (H-5501-60, Vector Labs).

### 2.7. RNAscope Imaging, Negative and Positive Controls, and Image Analysis

Following the completion of the RNAscope staining protocol, the slides were imaged at the Histocore facility, Karolinska Institute, Stockholm. Images were acquired on a Pannoramic MIDI slide scanner (software version 2.2.0.110942, 3DHISTECH Ltd., Budapest, Hungary), equipped with a 20× Plan-Apochromat objective lens. Each section underwent scanning across multiple focal planes (z-stacking) to ensure optimal focus throughout the tissue depth. The system was configured to automatically detect optimal focal planes, with focal limits set between positions 1600 to 2300, to accommodate variations in tissue thickness and to obtain detailed images suitable for analysis.

The scanned images were processed using CaseViewer (3DHISTECH Ltd., Budapest, Hungary) to generate multipage stitched TIFF files. This allowed for comprehensive analysis by combining individual image tiles into a seamless, high-resolution image of the entire tissue section.

To assess RNA integrity and validate the RNAscope Duplex assay procedure, we used two positive control probes targeting housekeeping genes Hs-PPIB (green) and POLR2A (red). Both of these probes produced distinct punctate staining in the tissue sections, demonstrating that the RNAscope procedure was successful and that the tissue RNA remained sufficiently intact for detection. The staining intensity for these positive controls was modest, which we attribute to the nature of our samples (frozen skin biopsies) and the lack of extensive pretreatment optimization for this novel application. Such modest signals are consistent with preserved RNA in frozen tissue sections, where some efficiency loss is expected. Importantly, even a moderate positive control signal is sufficient to confirm that the assay worked properly, and in our case, the presence of clearly visible PPIB and POLR2A dots indicates that our sample RNA quality was adequate and that the RNAscope assay conditions were appropriate (Figure S2).

We also included the ACD universal negative control probe targeting the bacterial dapB gene to assess non-specific background staining. This negative control slide showed no signal in the psoriatic skin tissue, indicating that background noise was minimal. The absence of the dapB signal confirms that any punctate staining observed with the VDR probe is not due to non-specific binding or assay artifacts but rather represents specific hybridization. This ensures that the RNAscope assay has low background noise and high specificity (Figure S2).

Together, these controls confirm the technical performance and specificity of our RNAscope assay. The clear background from the negative control ensures that the VDR signals we observed are specific, and the presence of even modest but discernible signals from the positive control shows that the assay worked correctly, and the RNA remained sufficiently intact. Consequently, the observed VDR punctate staining can be attributed to genuine transcript detection rather than nonspecific binding or technical artifacts.

Image analysis was conducted using Fiji (ImageJ package) software (version 1.54g), an open-source platform widely used for biological image analysis [29]. During analysis, the stained sections were evaluated for tissue morphology, staining intensity, and signal specificity.

Regions of interest (ROIs) within the epidermal and dermal layers were identified based on morphological features. The VDR expression within these ROIs was assessed using the semi-quantitative scoring system described in Section 2.8.

To ensure objectivity and eliminate potential bias, a blinding process was implemented by a fellow researcher who was not involved in the analysis. Randomly computer-generated codes were assigned to the images, concealing information regarding the patients’ ID-codes, treatment status (pre- or post-etanercept), and whether the images were from lesional or perilesional parts of the skin.

### 2.8. ROIs, Assessment, and Scoring of VDR Expression

VDR expression was assessed in both the epidermal and dermal layers of skin biopsies. In the epidermis, three ROIs were selected to evaluate the VDR expression across different layers:

1. Basal layer: The innermost layer of the epidermis, consisting of a single row of proliferating keratinocytes adjacent to the dermal-epidermal junction. Approximately ten basal cells were examined along a linear segment of the basal membrane for each analysis.
2. Stratum spinosum: Immediately above the basal layer, the stratum spinosum is composed of keratinocytes that have begun the differentiation process. In this region, an area encompassing approximately 8 to 10 cells in width and 8 to 10 cells in height was analyzed. This resulted in examining a block of cells, approximately totaling between 64 (8 × 8) to 100 (10 × 10) cells. The area started directly above the basal cells previously analyzed and extended upward into the stratum spinosum.
3. Apical layers: This region includes the outer layers of the viable epidermis. The analysis extended from the upper boundary of the previously analyzed stratum spinosum area upward through the stratum granulosum and continued until clear cell structures were no longer visible, which occurs within the stratum corneum. All cells within this vertical segment were included in the analysis, with their number varying depending on the thickness of these layers.
4. Dermis: The dermal region of interest was defined as the area immediately beneath the dermal-epidermal junction, focusing on the upper dermis where fibroblasts, endothelial cells, and infiltrating immune cells are located.

**Figure 1:**
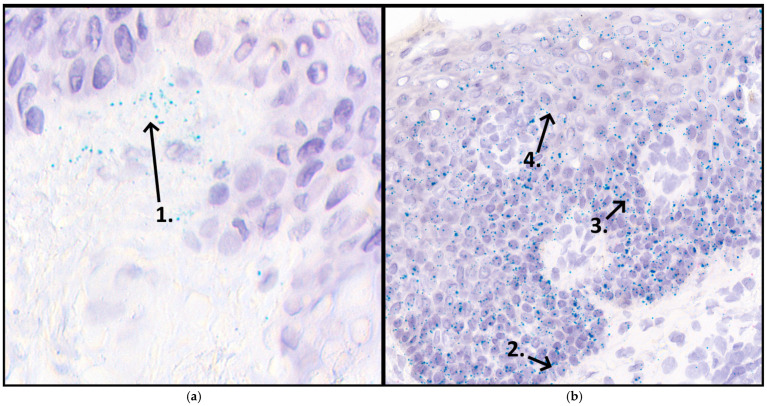
Vitamin D receptor (VDR) expression in lesional psoriasis skin after etanercept treatment, analyzed using RNAscope in situ hybridization (ISH). This image is presented as a representative illustration of the RNAscope methodology and is not intended for direct pre-post comparison or generalization. (**a**) The first panel highlights the dermis with the arrow (1) pointing to the area immediately beneath the dermal-epidermal junction. (**b**) The second panel displays arrows indicating the positions of the analyzed epidermal layers: basal layer (2), with the arrow pointing directly at the basal layer located at the dermal-epidermal junction; stratum spinosum (3), with the arrow pointing at the upper boundary of the stratum spinosum, far above the basal layer; Apical layers (4), with the arrow pointing past the stratum spinosum into the outer layers of the epidermis. The green punctate dots observed throughout the epidermis and dermis represent VDR. VDR expression was predominantly observed in the basal layer throughout the tissues.

A semi-quantitative scoring system was used to evaluate the levels of VDR expression within the defined ROIs. This scoring system was adapted from the guidelines provided in the RNAscope^®^ Assay manual [28]. The scoring was based on the number of punctate dots observed within individual cells, representing VDR mRNA transcripts detected by the assay (Figure 1).

For the epidermis and the individualized epidermal layers, the scoring system is as follows (Table 1):

In the dermis, due to the different cellular composition with varying expression levels throughout the tissue, a modified scoring system was used (Table 2), scaled appropriately to reflect the expression levels observed.

When possible, at least three areas within each ROI were analyzed per sample to ensure representative sampling and to minimize the impact of localized variability. The final score for each ROI was determined by calculating the average of the scores from the analyzed areas. For example, if the scores in three different areas of an ROI were 2, 3, and 1, the average score would be 2.0.

### 2.9. Data Quality Considerations

Several challenges affected data quality during the image analysis of VDR expression. Some images lacked sharpness due to technical limitations during scanning or inconsistencies in staining, making accurate counting of punctate dots difficult. Tissue damage during biopsy collection or handling resulted in incomplete or distorted regions. Missing data primarily resulted from histological limitations, including insufficient tissue in specific layers, tissue overlap, artifact presence, or contamination that interfered with visualization and complicated the identification of specific regions (Figure S3).

To maintain data integrity, we required that all three epidermal layers be intact and scorable for the total epidermis measure. Consequently, if one layer was damaged or unscoreable, we excluded that section from the total epidermis calculation. In contrast, we could still analyze any single layer individually, even if other layers in the same section were missing or damaged. This methodological choice resulted in fewer total-epidermis samples compared to individual-layer samples, as noted in the “Epidermal Layers—Individualized” section. The same tissue sections were used for scoring both the total and the individualized epidermal layers.

All images included were reviewed at least twice to ensure consistency, with discrepancies resolved through re-examination and consensus.

### 2.10. Statistical Analysis

All data were analyzed using R version 3.5.3 (The R Foundation for Statistical Computing, Vienna, Austria). Linear regression analysis was performed within each combination of patient and skin type (lesional or perilesional skin). The RNAscope scores served as the dependent variable, while treatment status was the independent variable (coded as 0 for before treatment and 1 for after treatment).

For assessing correlations between variables, Spearman’s correlation test was used. The Wilcoxon rank-sum test was used for two-sample comparisons. All tests were two-sided, and a *p*-value of <0.05 was considered statistically significant.

### 2.11. Ethical Considerations

This study received ethical approval from the regional ethics committee in Gothenburg (Dnr: 089-12) on 22 May 2012, with additional approvals by the Swedish Ethical Review Authority in 2014, 2016, and 2021. Conducted in accordance with the Declaration of Helsinki, this study ensured that all patients received standard clinical practice treatments, with no use of a placebo. Participants were fully informed about the study both orally and in writing before inclusion, and written consent was obtained for all skin biopsies and any other samples taken. OpenAI’s GPT engine 4o was used to assist with language editing and improvements.

## 3. Results

### 3.1. Baseline Parameters

The study included two adult male patients diagnosed with moderate to severe plaque psoriasis and one healthy control for comparison (Table 3).

Patient 1 (P1) presented with a PASI score of 10.8 and a DLQI score of 9.0, indicating moderate disease severity with a moderate impact on quality of life. In comparison, Patient 2 (P2) had a higher PASI score of 14.2 and a DLQI of 24.0, suggesting more severe skin involvement with a considerable impact on quality of life. The healthy control was free of skin disease, with no need for PASI or DLQI assessment or treatment plan.

### 3.2. Treatment Response

No serious adverse effects were reported by either patient during the treatment period. Both patients tolerated the etanercept regimen well, with no instances of complications that would warrant exclusion from the study.

The treatment outcomes for the two patients with psoriasis were evaluated clinically using the PASI and DLQI scores after 10–12 weeks of etanercept treatment (Table 4).

P1 achieved significant improvement with etanercept, with a PASI reduction of 89% and a DLQI score dropping to 0, indicating complete symptom relief and quality of life restoration. P2 had a moderate response, with a 27% PASI reduction and a 50% improvement in DLQI, though still experiencing notable disease impact.

### 3.3. VDR Expression Analysis

#### 3.3.1. Epidermis—Total VDR Score

**Figure 2:**
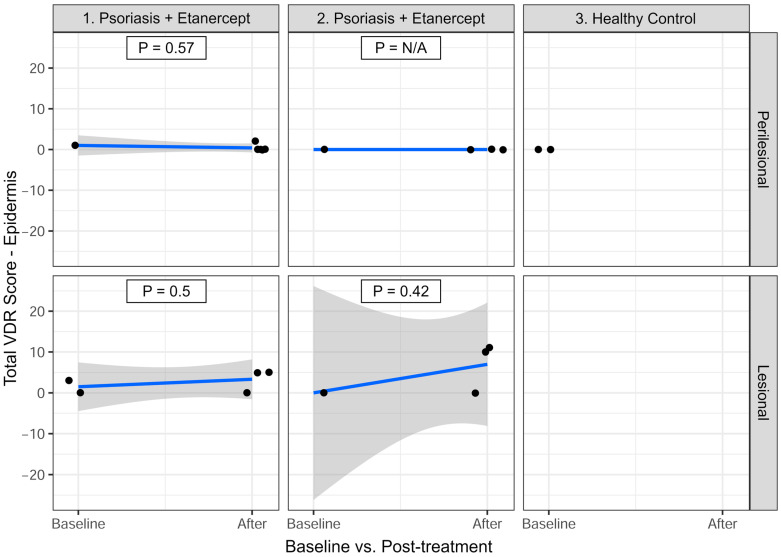
Total vitamin D receptor (VDR) expression in the epidermis (all layers summarized) at baseline and after 10–12 weeks of etanercept treatment. VDR expression was assessed using a 0–4 scoring system across all three epidermal layers analyzed: basal, stratum spinosum, and apical, providing a maximum score of 12 per biopsy section. The graphs were generated using R, and identical or nearly identical values may naturally appear superimposed in the output.

As seen in Figure 2, for P1, in perilesional skin, the mean VDR expression decreased from 1.00 at baseline to 0.40 after treatment (Estimate = −0.60; *p* = 0.57; n = 6). In lesional skin, the mean VDR expression increased from 1.50 at baseline to 3.33 after treatment (Estimate = 1.83; *p* = 0.50; n = 5).

For P2, in perilesional skin, VDR expression remained at 0.00 before and after treatment (Estimate = 0.00; *p* = N/A; n = 4). In lesional skin, VDR expression increased from 0.00 at baseline to 7.00 after treatment (Estimate = 7.00; *p* = 0.42; n = 4).

Neither patient exhibited statistically significant changes in VDR expression in the epidermis following etanercept treatment (*p* > 0.05).

#### 3.3.2. Dermis

**Figure 3:**
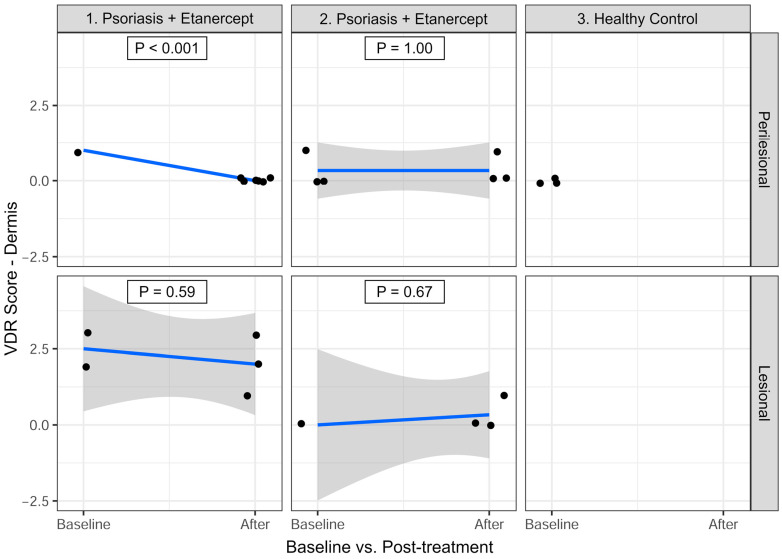
Vitamin D receptor (VDR) expression in the dermis at baseline and after 10–12 weeks of etanercept treatment. VDR expression was assessed using a 0–3 scoring system, providing a maximum possible score of 3 per biopsy section. The graphs were generated using R, and identical or nearly identical values may naturally appear superimposed in the output.

As seen in Figure 3, for P1, VDR expression in the perilesional dermis showed a significant decrease after treatment, from a mean of 1.00 at baseline to 0.00 after treatment (Estimate = −1.00; *p* < 0.001). In the lesional dermis, the mean VDR expression decreased slightly from 2.50 to 2.00 (Estimate = −0.50; *p* = 0.59).

For P2, the VDR expression in the perilesional dermis remained unchanged at 0.33 before and after treatment (Estimate = 0.00; *p* = 1.00). In the lesional dermis, it increased slightly from 0.00 to 0.33 (Estimate = 0.33; *p* = 0.67).

Only P1 demonstrated a statistically significant decrease in VDR expression in the perilesional dermis after treatment (*p* < 0.001). No other statistically significant changes were observed in dermal VDR expression for either patient (*p* > 0.05).

#### 3.3.3. Epidermal Layers—Individualized

VDR expression was assessed separately in the basal layer, stratum spinosum, and apical layer of the epidermis to investigate potential layer-specific effects of etanercept treatment.

In the perilesional skin, for P1, in the basal layer, the mean VDR expression increased slightly from 0.00 to 0.50 (Estimate = 0.50, *p* = 0.60; n = 7). In the stratum spinosum, VDR expression decreased from 1.00 to 0.50 (Estimate = −0.50, *p* = 0.60; n = 7). In the apical layer, VDR expression remained at 0.00 with no change (Estimate = 0.00, *p* = N/A; n = 6).

For P2, in the basal layer, VDR expression decreased from 0.67 to 0.00 (Estimate = −0.67, *p* = 0.37; n = 6). In the stratum spinosum, it decreased from 0.33 to 0.00 (Estimate = −0.33, *p* = 0.37; n = 6). In the apical layer, VDR expression remained at 0.00 with no change (Estimate = 0.00, *p* = N/A, n = 4).

In the lesional skin, both patients demonstrated increases in VDR expression across all epidermal layers after treatment, but these changes were not statistically significant. For P1, in the basal layer, VDR expression increased from 1.00 to 1.33 (Estimate = 0.33, *p* = 0.79; n = 5). In the stratum spinosum, it increased from 0.50 to 1.33 (Estimate = 0.83, *p* = 0.44; n = 5). In the apical layer, VDR expression increased from 0.00 to 0.67 (Estimate = 0.67, *p* = 0.22; n = 5).

For P2, in the basal layer, VDR expression increased from 0.00 to 2.67 (Estimate = 2.67, *p* = 0.42; n = 4). In the stratum spinosum, it increased from 0.00 to 2.33 (Estimate = 2.33, *p* = 0.43; n = 4). In the apical layer, VDR expression increased from 0.00 to 2.00 (Estimate = 2.00, *p* = 0.42; n = 4).

When analyzing the images, higher VDR expression was most often observed in the basal layer compared to the stratum spinosum and apical layers. Overall, the individualized analysis of epidermal layers revealed no statistically significant alterations in VDR expression in either perilesional or lesional skin after etanercept therapy (Figures S4–S6).

## 4. Discussion

This prospective intervention study is novel in applying RNAscope to successfully analyze VDR expression in psoriatic skin tissue following etanercept treatment. To the best of our knowledge, this is the first time this method has been utilized in this context.

Our study used RNAscope, which offers high sensitivity and specificity for detecting RNA in situ, making it a powerful tool for gene expression analysis in skin biopsies. However, before this study, its utility in patients with psoriasis remained unexplored. Our findings demonstrate that RNAscope can reliably assess VDR expression in psoriatic skin biopsies, providing a foundation for future investigations with larger cohorts.

RNAscope specifically detects VDR mRNA, providing insights at the transcriptional level. In contrast, previous studies employed immunohistochemistry to assess VDR protein levels, offering information on protein localization and abundance [23,24,25,26,27]. This difference might contribute to varying findings, as mRNA expression does not always directly correlate with protein expression due to post-transcriptional and post-translational regulatory mechanisms [30].

We observed that etanercept treatment led to a significant decrease in VDR expression in the perilesional dermis of one psoriasis patient. This was accompanied by substantial clinical improvement, as evidenced by a large reduction in PASI and complete remission of DLQI. In contrast, no statistically significant changes in VDR expression were detected in the lesional epidermis or dermis for either patient, although there was a trend toward increased VDR expression in the lesional epidermis post-treatment in both patients independently of the treatment response. However, given the very small sample size, these results remain highly preliminary, cannot be generalized, and should be interpreted with caution.

Regarding the results, while Milde et al. [23] did not analyze pre- and post-treatment biopsies, they reported an overall increase in VDR expression in psoriatic lesions compared to perilesional skin, which is consistent with our observation of a possible upregulation of VDR in psoriatic lesions.

However, our results contrast with those of Kim et al., Visconti et al., and Elgarhy et al., who found decreased VDR expression in psoriatic lesions compared to normal skin [24,25,27]. Notably, Elgarhy et al. observed that VDR expression increased in psoriatic lesions following narrow-band UVB therapy, whereas we detected a significant decrease in VDR expression in the perilesional dermis after etanercept treatment [27]. These discrepancies might be attributed to differences in treatment modalities, as UVB therapy and TNF-α inhibition may have distinct effects on VDR expression. Furthermore, methodological differences, such as variations in detection methods, patient demographics, and the specific layers of skin analyzed, could contribute to the inconsistent findings regarding VDR expression.

Comparing our findings with those of Zanghaneh et al., who studied VDR expression on peripheral blood mononuclear cells (PBMCs) in patients with psoriasis treated with etanercept, reveals notable differences [31]. While they observed higher VDR expression on CD14⁺ monocytes at baseline with no significant changes during treatment, we found a significant decrease in VDR expression in the perilesional dermis post-etanercept therapy. This discrepancy may be attributed to the analysis of different materials (blood vs. skin) and suggests that etanercept may affect VDR expression differently in local skin cells compared to circulating immune cells. Additionally, the distinct methodologies (RNAscope vs. flow cytometry) might contribute to variations in detected VDR expression. An interesting area for further research would be to evaluate the potential correlations between VDR expression in blood and skin tissues, and between serum vitamin D levels and VDR expression.

A methodological consideration in our study is the use of chromogenic agents instead of a fluorescent approach. Although chromogenic detection allows for the visualization of up to two mRNA targets, fluorescent RNAscope offers higher sensitivity and a broader dynamic range, which can improve the detection of low-abundance transcripts like VDR mRNA [32]. Fluorescent methods also enable multiplexing beyond two targets, facilitating a more comprehensive analysis of gene expression profiles in psoriatic skin [28]. Additionally, fluorescence can provide better quantification and resolution, aiding in the precise localization of mRNA within tissue sections. The usage of fluorescent RNAscope in future studies could enhance the sensitivity and specificity of VDR expression analysis, offering deeper insights into the molecular mechanisms involved [33].

Furthermore, RNAscope employs a proprietary ‘double Z’ probe design, which requires both probes to hybridize in tandem before signal amplification, minimizing background noise [28]. Accurate results hinge on careful tissue handling, including rapid processing, consistent fixation times, and fixatives that preserve RNA integrity [34]. Minimizing the time from tissue acquisition to fixation, choosing appropriate fixatives, and standardizing fixation duration are essential to maintain consistent transcript quality [35]. Equally crucial is ensuring uniform sample handling, including rapid freezing and controlled processing temperatures. These steps reduce the risk of RNA degradation and variability, particularly in the delicate context of frozen skin biopsies. RNAscope can also circumvent common pitfalls of immunohistochemistry, such as antibody specificity and epitope variability [35]. This methodological precision makes it particularly valuable for studying mRNA transcripts in complex tissues like psoriatic skin.

Regarding the histological limitations, potential reasons include variable biopsy orientation during cryosectioning, uneven tissue thickness, or suboptimal embedding techniques leading to overlapping or missing layers. Consistent tissue orientation, careful calibration of the cryostat, and standardized embedding protocols may mitigate these issues. Future studies should incorporate quality checkpoints, such as preliminary test cuts viewed under a bright-field microscope, to ensure complete, intact layers for more reliable VDR quantification.

Analyzing different epidermal layers is important because VDR expression seems to vary across these layers. Milde et al. and Chandra et al. both reported significant VDR presence in the basal layer of the epidermis, which is crucial for keratinocyte proliferation and differentiation [23,26]. Similarly, our study observed that VDR expression was most prominent in the basal layer and decreased toward the outer layers of the epidermis, with minimal but detectable levels even in the stratum corneum.

This study has several limitations. The most significant is the small sample size of only two patients and one healthy control, which limits the statistical power and generalizability of the findings. Additionally, technical challenges during tissue processing and image analysis may have introduced variability that could affect the results. Furthermore, we assessed VDR mRNA expression without evaluating corresponding protein levels, which may not directly correlate due to post-transcriptional regulation and could be of interest in future studies.

Building upon these findings, conducting larger-scale studies with more participants would enhance statistical power and improve the generalizability of the results. By including a greater number of patients and controls, future studies could validate the preliminary observations made in this study and provide more robust statistical analyses. A larger cohort would also allow for subgroup analyses.

The use of artificial intelligence (AI) for the analysis of biopsy sections could provide more objective and consistent quantification of VDR expression. AI algorithms, trained on extensive datasets, could reduce observer bias and improve accuracy in scoring, enabling high-throughput analysis and potentially uncovering subtle patterns in VDR expression that may be missed by manual evaluation.

Additionally, investigating the correlation between changes in VDR expression and clinical parameters like PASI and DLQI scores in a larger cohort could reveal meaningful associations. Such correlations might help determine whether VDR expression could serve as a biomarker for disease severity or treatment response, resulting in more personalized treatment strategies.

## 5. Conclusions

This study demonstrates the feasibility of using RNAscope, a novel RNA ISH technique, to analyze VDR expression in psoriatic skin tissue following etanercept treatment. Our results indicate that etanercept may influence VDR expression, showing a significant decrease in the perilesional dermis of one patient and a trend toward increased expression in the lesional epidermis in both patients, independently of the treatment response. However, due to the small sample size, these findings are preliminary and should be interpreted with caution. There is a clear need for larger cohort studies to validate these observations and to address the lack of consensus in the literature regarding VDR expression in psoriasis. Further research is essential to deepen our understanding of the role of VDR in psoriasis pathogenesis and to determine its potential as a therapeutic target.

## Figures and Tables

**Table 1:** Semi-quantitative scoring system for vitamin D receptor (VDR) expression in the epidermis and the individualized epidermal layers.

Score ^1^	Expression Level (Dots Per Cell)
0	No expression or <1 dot per 10 cells
1	1–2 dots per cell
2	3–5 dots per cell
3	6–10 dots per cell
4	>10 dots per cell

^1^ The scoring system was adapted according to the RNAscope^®^ Assay manual guidelines.

**Table 2:** Semi-quantitative scoring system for vitamin D receptor (VDR) expression in the dermis.

Score ^1^	Expression Level (Dots Per Cell)
0	No expression or almost none
1	A few scattered expressions
2	Moderate expression, some clusters
3	Widespread expression, frequent clusters

^1^ The scoring system was adapted according to the RNAscope^®^ Assay manual guidelines.

**Table 3:** Baseline parameters, including Psoriasis Area Severity Index (PASI) and Dermatology Life Quality Index (DLQI) scores of patients with psoriasis before treatment with etanercept, and a healthy control. Some healthy control variables are not applicable (N/A).

Variable.	Patient 1 **	Patient 2 **	Healthy Control
Age (years) *	65	41	68
Sex	Male	Male	Male
Skin disease	Psoriasis	Psoriasis	N/A
Treatment	Etanercept	Etanercept	N/A
Biopsied area	Gluteus	Gluteus	Gluteus
PASI	10.8	14.2	N/A
DLQI	9.0	24.0	N/A

* Age at inclusion in the study. ** Referred to as P1 and P2 in the following sections.

**Table 4:** Treatment response was measured by changes in Psoriasis Area Severity Index (PASI) and Dermatology Life Quality Index (DLQI) scores after 10–12 weeks of etanercept therapy in patients with psoriasis. Variables for the healthy control are not applicable (N/A). Arrows (↓) indicates reduction (improvement) in PASI and DLQI scores.

Variable	Patient 1 *	Patient 2 *	Healthy Control
PASI	1.2	10.4	N/A
DLQI	0	12.0	N/A
Difference in PASI, %	↓ 89%	↓ 27%	N/A
Difference in DLQI, %	↓ 100%	↓ 50%	N/A

* Referred to as P1 and P2 in the following sections.

## Data Availability

Data supporting the study’s findings will be made available by the corresponding author upon reasonable request.

## Supplementary Materials

The following supporting information can be downloaded at https://www.mdpi.com/article/10.3390/cimb47050311/s1.
