# Supplementary figures and images for: A Novel Application of RNA In Situ Hybridization in the Analysis of Vitamin D Receptor Expression in Psoriatic Skin Tissue Following Etanercept Treatment
Source: Curr Issues Mol Biol. 2025 Apr 28;47(5):311. doi: 10.3390/cimb47050311 (PMC12110436; doi:10.3390/cimb47050311)

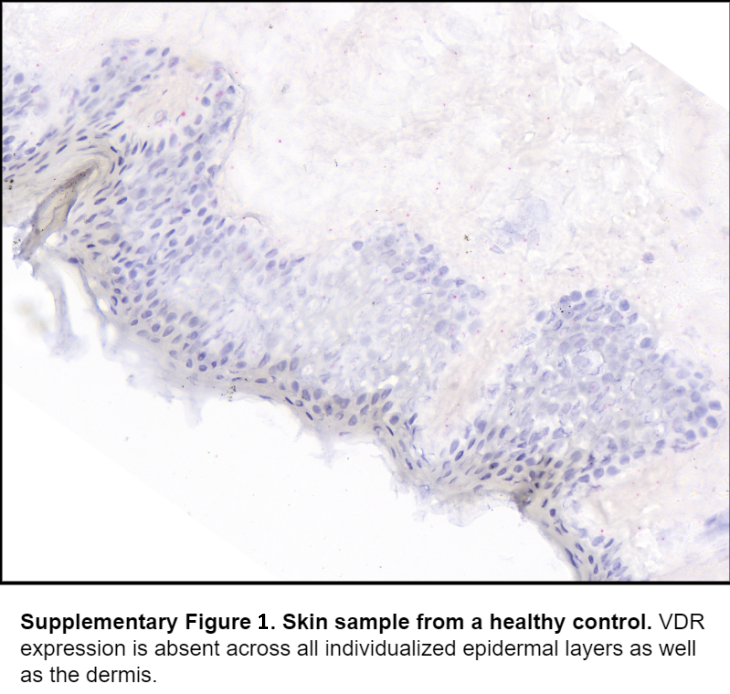

Supplement: Supplementary file 1 [file cimb-47-00311-s001.zip › Figure S1 í¬ Healthy Control.png]

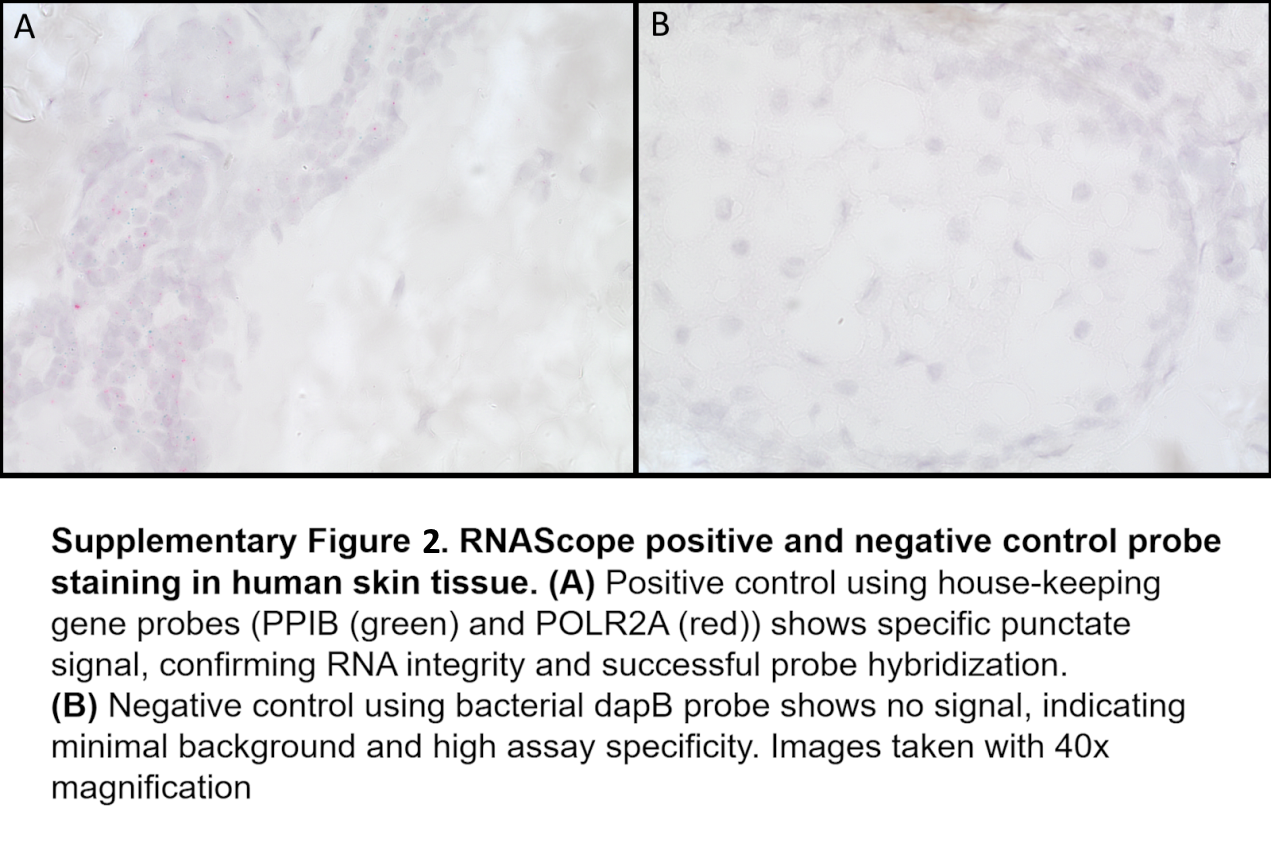

Supplement: Supplementary file 1 [file cimb-47-00311-s001.zip › Figure S2 í¬ Positive and Negative Control.png]

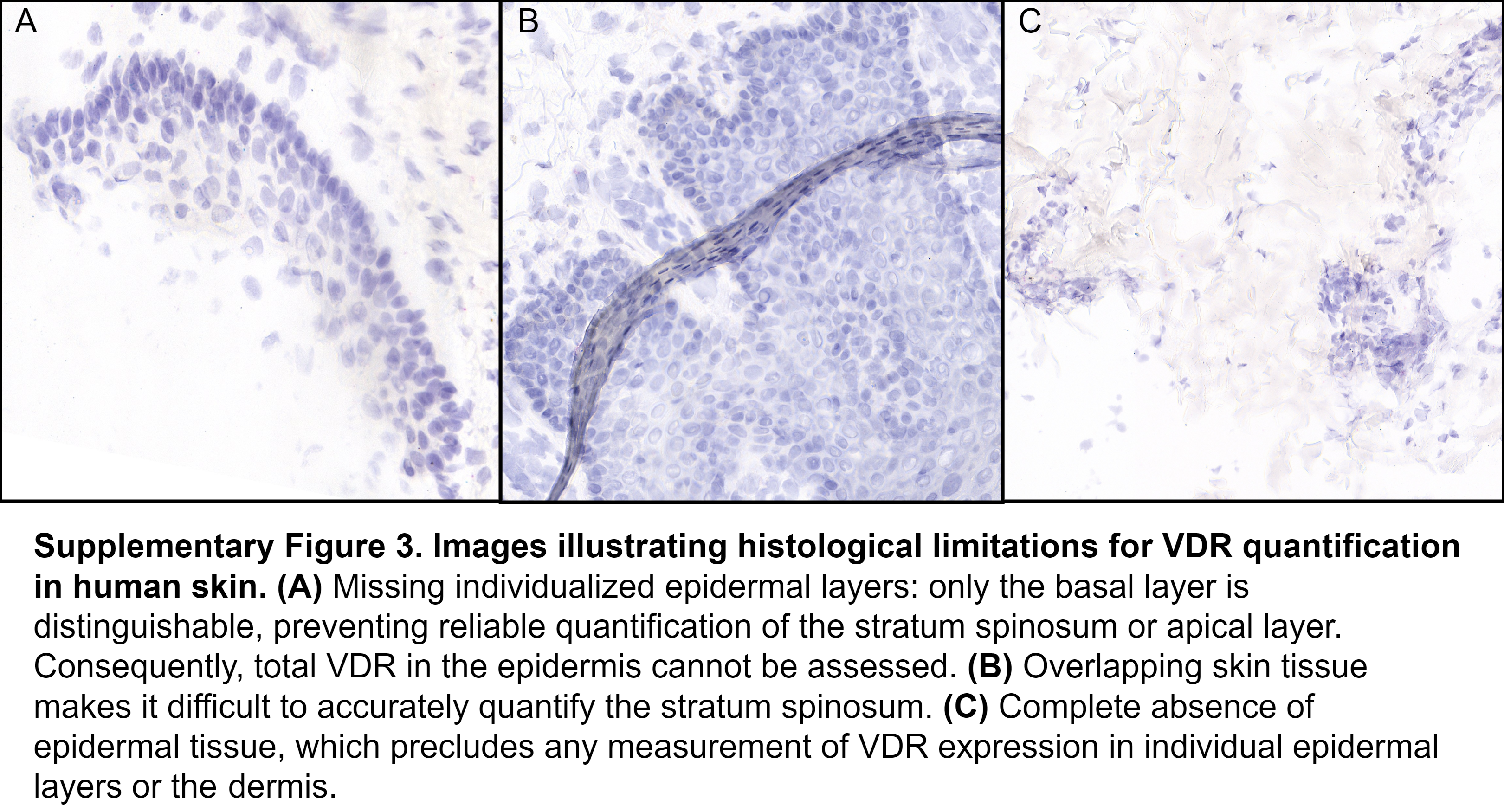

Supplement: Supplementary file 1 [file cimb-47-00311-s001.zip › Figure S3 í¬ Histological Limitations.png]

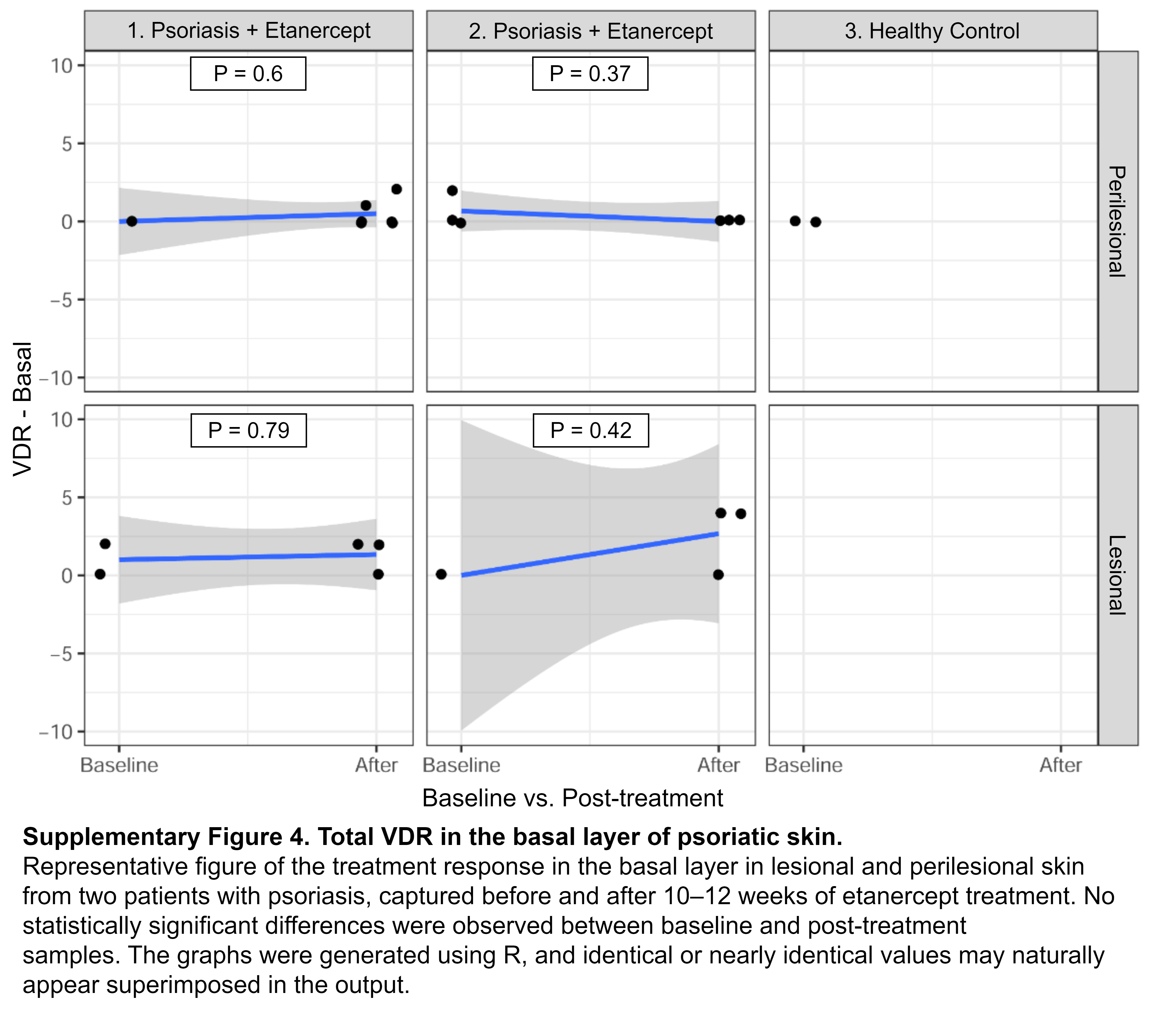

Supplement: Supplementary file 1 [file cimb-47-00311-s001.zip › Figure S4 í¬ Individualized VDR in Basal.png]

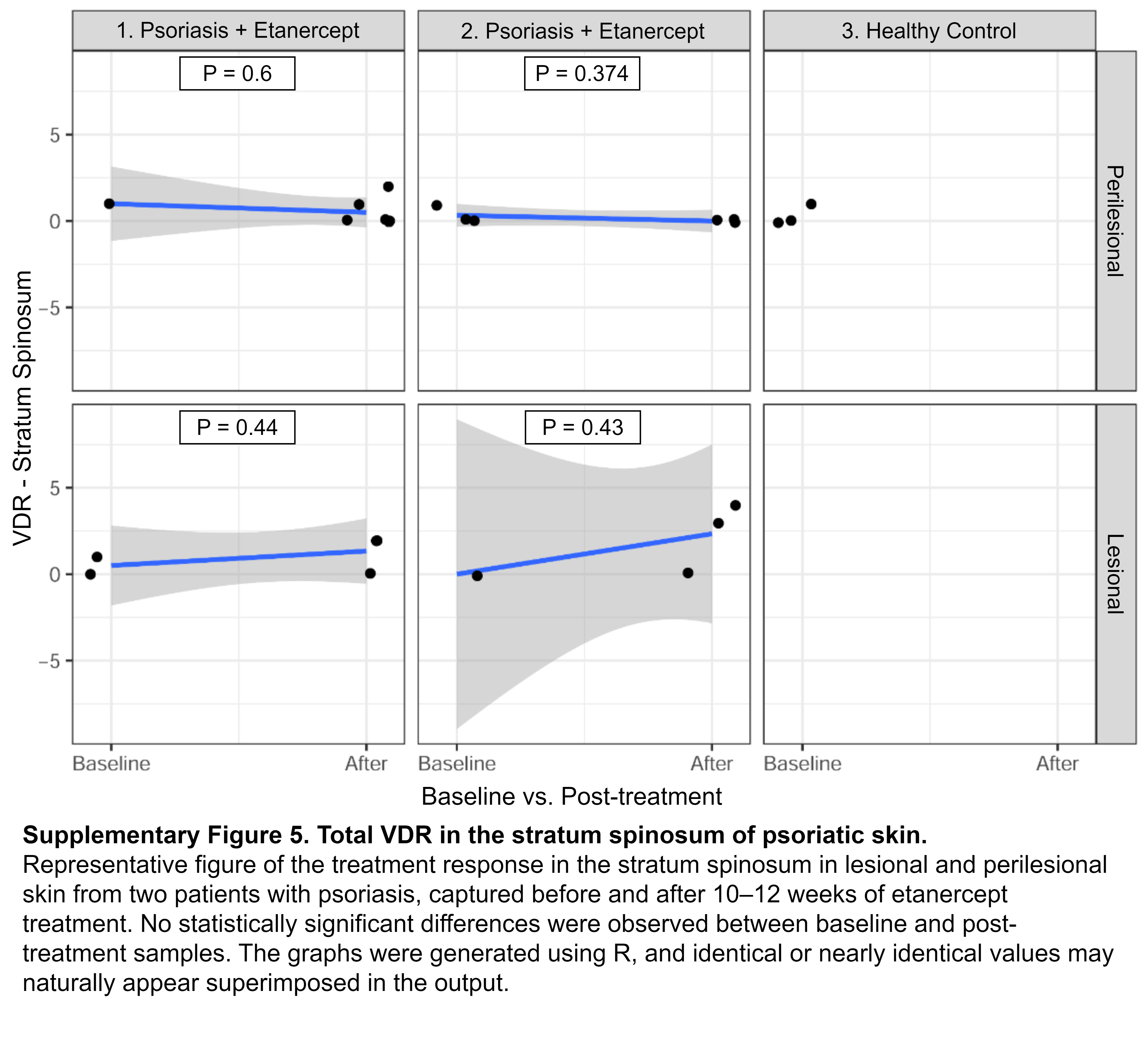

Supplement: Supplementary file 1 [file cimb-47-00311-s001.zip › Figure S5 í¬ Individualized VDR in Stratum Spinosum.png]

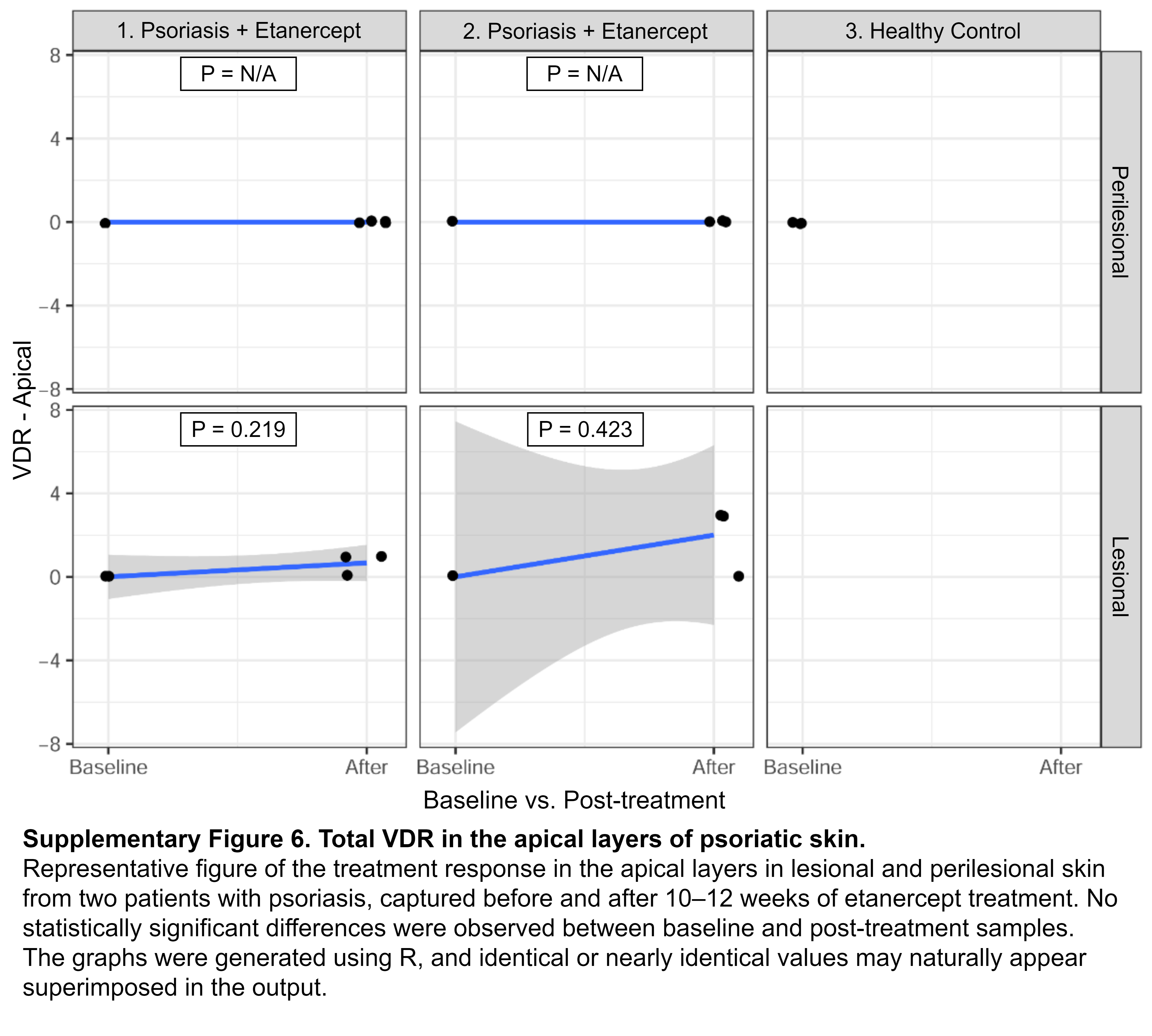

Supplement: Supplementary file 1 [file cimb-47-00311-s001.zip › Figure S6 í¬ Individualized VDR in Apical.png]
